# Evolutionarily conserved amino acids in MHC-II mediate bat influenza A virus entry into human cells

**DOI:** 10.1371/journal.pbio.3002182

**Published:** 2023-07-06

**Authors:** Okikiola M. Olajide, Maria Kaukab Osman, Jonathan Robert, Susanne Kessler, Lina Kathrin Toews, Thiprampai Thamamongood, Jacques Neefjes, Antoni G. Wrobel, Martin Schwemmle, Kevin Ciminski, Peter Reuther

**Affiliations:** 1 Institute of Virology, Medical Center – University of Freiburg, Freiburg, Germany; 2 Faculty of Medicine, University of Freiburg, Freiburg, Germany; 3 Spemann Graduate School of Biology and Medicine, University of Freiburg, Freiburg, Germany; 4 Faculty of Biology, University of Freiburg, Freiburg, Germany; 5 Virology and Cell Technology Laboratory, National Center for Genetic Engineering and Biotechnology (BIOTEC), National Science and Technology Development Agency, Khlong Nueng, Khlong Luang District, Pathum Thani, Thailand; 6 Department of Cell and Chemical Biology, Oncode Institute, Leiden University Medical Center, Leiden, the Netherlands; 7 Structural Biology of Disease Processes Laboratory, The Francis Crick Institute, London, United Kingdom; University of Wisconsin-Madison, UNITED STATES

## Abstract

The viral hemagglutinins of conventional influenza A viruses (IAVs) bind to sialylated glycans on host cell surfaces for attachment and subsequent infection. In contrast, hemagglutinins of bat-derived IAVs target major histocompatibility complex class II (MHC-II) for cell entry. MHC-II proteins from various vertebrate species can facilitate infection with the bat IAV H18N11. Yet, it has been difficult to biochemically determine the H18:MHC-II binding. Here, we followed a different approach and generated MHC-II chimeras from the human leukocyte antigen DR (HLA-DR), which supports H18-mediated entry, and the nonclassical MHC-II molecule HLA-DM, which does not. In this context, viral entry was supported only by a chimera containing the HLA-DR α1, α2, and β1 domains. Subsequent modeling of the H18:HLA-DR interaction identified the α2 domain as central for this interaction. Further mutational analyses revealed highly conserved amino acids within loop 4 (N149) and β-sheet 6 (V190) of the α2 domain as critical for virus entry. This suggests that conserved residues in the α1, α2, and β1 domains of MHC-II mediate H18-binding and virus propagation. The conservation of MHC-II amino acids, which are critical for H18N11 binding, may explain the broad species specificity of this virus.

## Abstract 2

Bat influenza A viruses use major MHC-II for cell entry; this study shows that highly conserved amino acids within MHC-II are key for infection and provide an explanation for the ability of these viruses to target MHC-II from a broad range of vertebrates, including humans.

## Introduction

Zoonotic transmission of viruses represents a constant threat to global health. Bats play an important role as reservoir hosts for diverse, potentially deadly viral pathogens [1–4]. However, until recently, bats were not recognized as a reservoir for influenza A viruses (IAVs); rather, all IAV strains were believed to have originated from wild waterfowls [5]. This notion was challenged by the discovery of the genome sequences of 2 novel IAV strains, H17N10 and H18N11, in New World bats [6–8]. Although these bat IAVs essentially resemble conventional IAVs, their surface glycoproteins (H17/18 and N10/11) differ fundamentally in function from those of conventional IAVs despite structural similarity. While the hemagglutinins of conventional IAV (H1 to H16) mediate attachment and entry via binding to sialic acid residues, both H17 and H18 are unable to bind sialylated glycans [8–10] and instead utilize major histocompatibility complex class II (MHC-II) molecules for cell entry [11].

MHC-II molecules are heterodimeric transmembrane proteins essential for adaptive immune responses as they present antigenic peptides of extracellularly derived proteins on the surface of professional antigen presenting cells to CD4^+^ T cells [12–14]. They consist of alpha (α)- and beta (β)-chains made of membrane-proximal barrel-shaped, immunoglobulin (Ig)-like α2 and β2 domains and juxtaposed membrane-distal domains (α1 and β1), which contribute almost equally to the formation of the peptide-binding groove [13,15,16]. MHC-II molecules fold in the endoplasmic reticulum, from which they are transported with the help of the invariant chain to late endosomal compartments, where the invariant chain is degraded and MHC-II loaded with peptides [13,14]. This peptide loading is facilitated by a chaperone, the nonclassical MHC-II molecule DM (in human HLA-DM) [14,17]. HLA-DM resides in the late endosomal compartments and shares high structural similarity with classical MHC-II molecules but does not have a functional peptide-binding groove [14,18]. After binding a peptide, MHC-II molecules are trafficked to the plasma membrane [19].

The H18 binding site on MHC-II is unknown. However, classical MHC-II molecules from all vertebrate species tested to date enable H18-mediated infection [11], suggesting that highly conserved MHC-II residues facilitate viral entry. Here, we sought to define these residues through a mutational approach in which we generated chimeric MHC-II molecules from permissive classical HLA-DR and nonpermissive nonclassical HLA-DM molecules. We identified conserved residues within the α2 domain of HLA-DR as key for H18N11 infection.

## Results

### The β2 domain of HLA-DR is dispensable for H18-mediated entry

**Fig 1:**
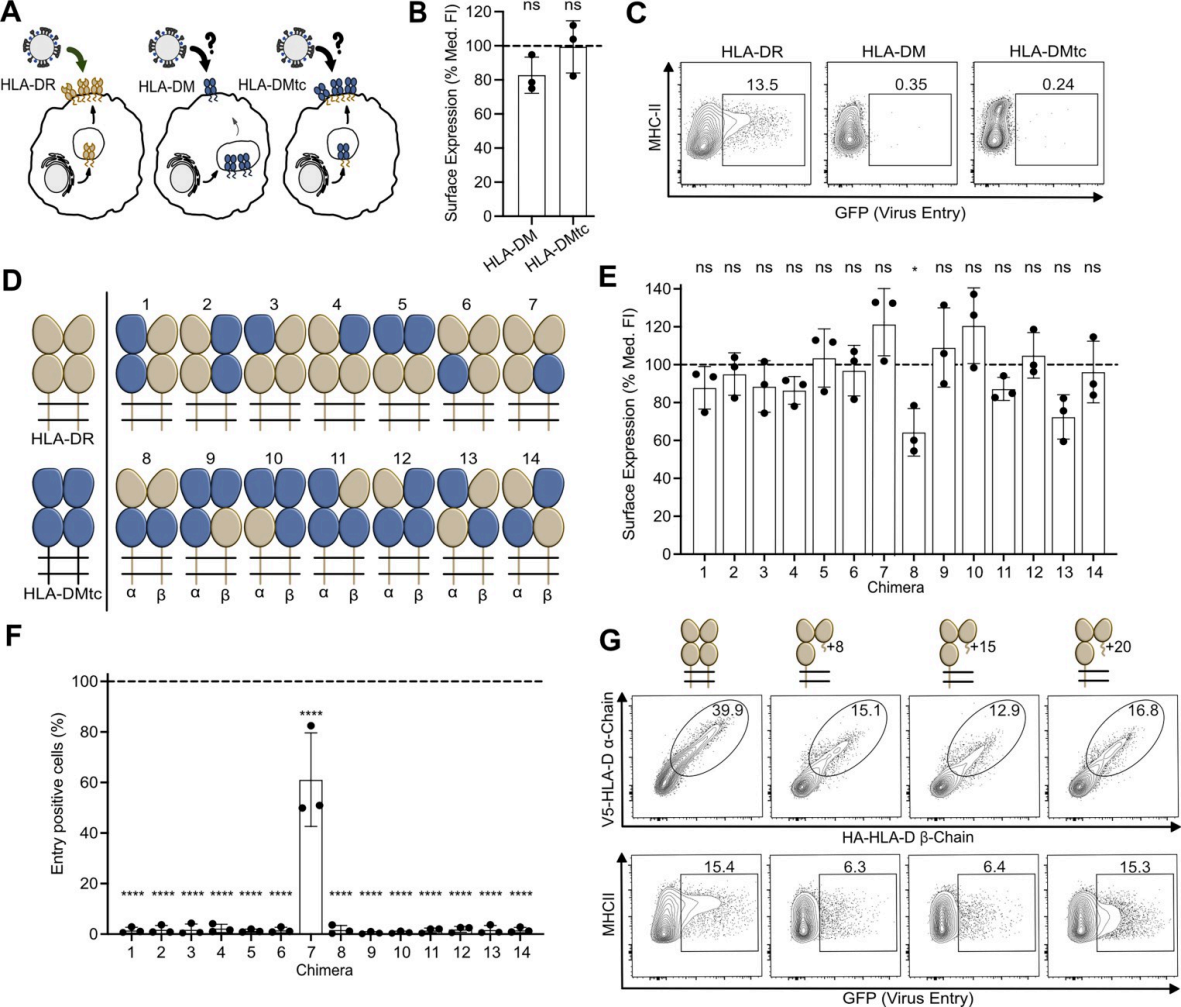
The β2 domain of HLA-DR is not required for H18-mediated entry. **(A)** Schematic representation of the expression pattern of HLA-DR, HLA-DM, and HLA-DM_tc_ (HLA-DM with transmembrane and cytoplasmic domain replaced by those of HLA-DR). While cells expressing HLA-DR support entry of bat IAV, the ability of cells expressing HLA-DM or HLA-DM_tc_ to support entry of bat IAV is to be tested. **(B)** Flow cytometric analysis of the surface expression of HLA-DM and HLA-DM_tc_. HEK293T cells were transfected with plasmids encoding the respective V5-tagged MHC-II α- and HA-tagged MHC-II β-chains. The bar graph depicts the median fluorescent intensity of the β-chain determined from cells that fall into the α-chain and β-chain double positive gate (S1B Fig). Values were normalized to wild-type HLA-DR (dashed line). Underlying data: S1 Data. **(C)** Susceptibility of HEK293T cells transfected with the respective MHC-II complexes to infection with VSV-H18 encoding a GFP reporter protein. Infected, GFP-positive cells among the MHC-II-expressing population were quantified by flow cytometry. Numbers within the plots indicate percentages of cells falling into the respective gates. **(D)** Illustration of the HLA-DR/HLA-DM chimeric MHC-II complexes used in (E) and (F). HLA-DR-derived domains are colored in beige; HLA-DM-derived domains are depicted in dark blue. **(E)** Flow cytometric analysis of surface expression of the respective chimeric MHC-II complexes as described in (B). Underlying data: S1 Data. **(F)** Susceptibility of cells expressing chimeric MHC-II complexes to VSV-H18 infection relative to HLA-DR-expressing cells (dashed line). Underlying data: S1 Data. **(G)** Quantification of HEK293T cells expressing HLA-DR complexes comprising the illustrated truncations of the β2 domain (upper row) and their susceptibility to VSV-H18 infection (lower row). Numbers within the flow cytometry plots indicate percentages of cells falling into the respective gates. Flow cytometry plots are representatives of 3 independent experiments. Error bars represent SD. For statistical analysis, one-way ANOVA followed by Dunnett test was performed. * *P* < 0.05, **** *P* < 0.0001, ns, not significant. HLA-DR, human leukocyte antigen DR; IAV, influenza A virus; MHC-II, major histocompatibility complex class II.

To test whether the intracellular nonclassical MHC-II molecule HLA-DM can support H18-mediated cell entry, we generated a chimeric HLA-DM with its transmembrane and cytoplasmic domains swapped for those of HLA-DR (HLA-DMtc) (Figs 1A, S1A and S1B). Following transfection of human embryonic kidney (HEK) 293T cells with plasmids coding for HLA-DR, HLA-DM, and HLA-DMtc, we detected efficient surface expression of HLA-DMtc similar to that of HLA-DR by flow cytometry (Figs 1B and S2, S1 Data). However, only cells expressing HLA-DR but neither wild-type HLA-DM nor HLA-DMtc were able to support entry of a GFP-encoding vesicular stomatitis virus whose glycoprotein was replaced by H18 (VSV-H18) [20] (Fig 1C), suggesting that the HLA-DM structure is incompatible with H18 binding.

We then generated further HLA-DR/DM chimeras by replacing individual domains of the α- and β-chains of HLA-DR with the corresponding domains of HLA-DM (Figs 1D, S1A and S1B). As shown in Fig 1E and S1 Data, all chimeras were expressed at the cell surface at levels comparable to HLA-DR except for chimeras 8 and 13, which showed lower cell surface expression. Irrespective of cell surface expression levels, only chimera 7, comprising the α1, α2, and β1 domains of HLA-DR in assembly with the β2 of HLA-DM, allowed infection with VSV-H18 (Figs 1F and S3A, S1 Data), suggesting that the β2 domain of HLA-DR is dispensable for virus entry. To test this, we coexpressed the full-length HLA-DR α-chain with truncated versions of the HLA-DR β-chain consisting of the β1 domain with short (8, 15, or 20 residues) fragments of the β2 domain retained at its C-terminus (Fig 1G). These truncated heterodimers accumulated at the cell surface, albeit to a lesser extent than intact HLA-DR, and supported VSV-H18 infection (Fig 1G).

### The α2 domain of HLA-DR is key for H18-mediated entry

**Fig 2:**
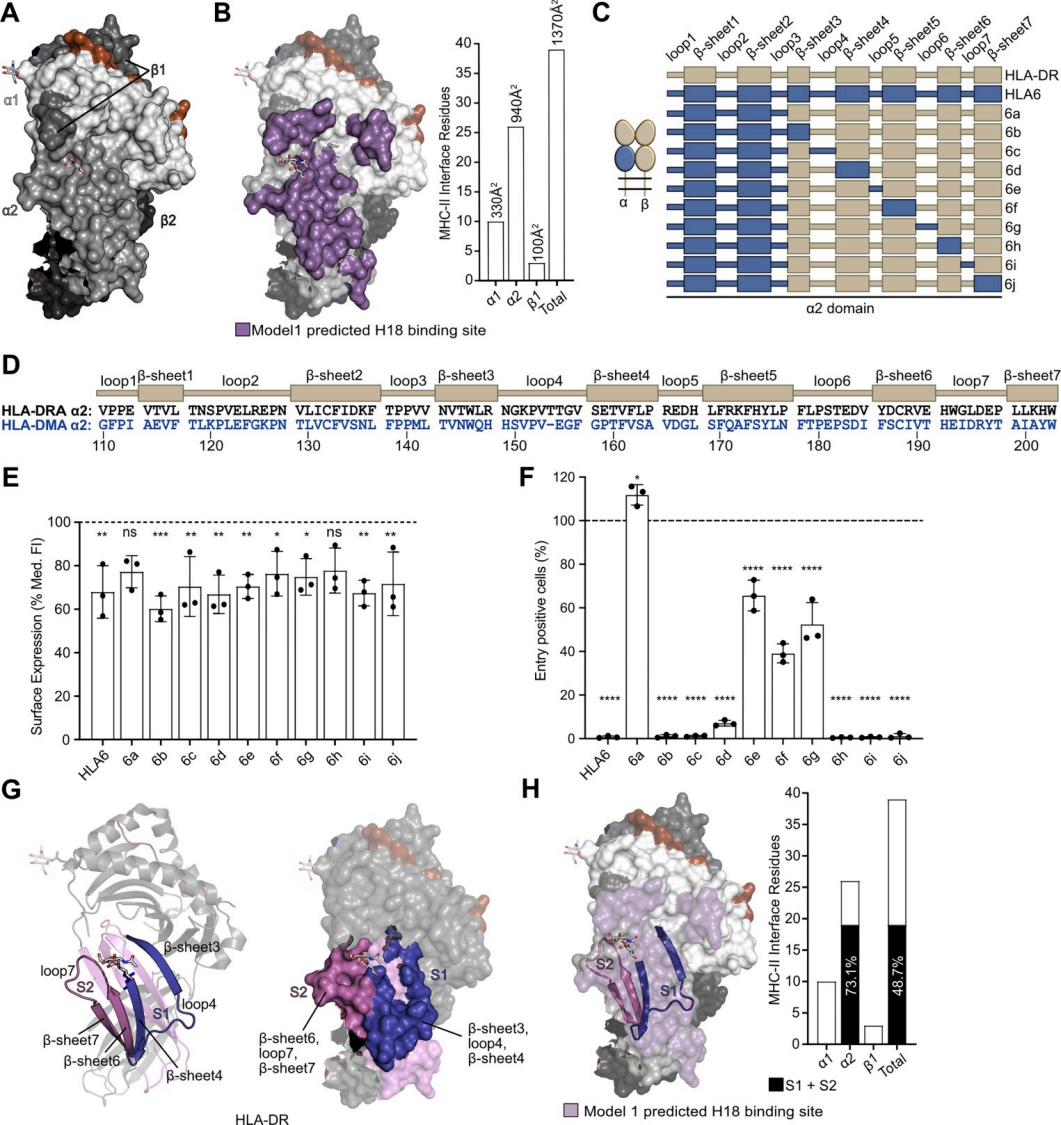
The α2 domain of HLA-DR is crucial for H18-mediated entry. **(A)** Surface representation of a crystal structure (PDB code: 1DLH) [41] of HLA-DR bound to an antigenic peptide. The α1, α2, β1, and β2 domains are colored in different shades of gray (light to dark in the order indicated), and the peptide is colored in brown. **(B)** Surface representation of HLA-DR with residues predicted by model 1 to form the H18-binding interface in purple. The bar graph depicts the number of HLA-DR residues that contribute to H18 binding within the indicated domains and in total. Numbers above the bars show contribution of the domains to the predicted interacting surface area. **(C)** Illustration of the chimeric HLA-DR used in (E) and (F) with the indicated motifs of the α2 domain replaced by their HLA-DM-derived counterparts. HLA-DR-derived subdomains are colored in beige; HLA-DM-derived subdomains are colored in dark blue. **(D)** Amino acid alignment of the α2 domain of HLA-DR (black) and HLA-DM (blue), highlighting the amino acid sequences of loops and β-sheets shown in (C). **(E)** Flow cytometric analysis of the surface expression of the indicated MHC-II complexes transiently expressed in HEK293T cells. The bar graph depicts the median fluorescence intensity of the β-chain of cells that are double positive for the α- and β-chain (see S1C Fig), normalized to HLA-DR (dashed line). Underlying data: S2 Data. **(F)** Flow cytometric quantification of VSV-H18-infected, GFP-positive HEK293T cells transiently expressing the respective chimeric MHC-II complexes. The percentage of GFP-positive cells is normalized to that of infected, HLA-DR-expressing cells (dashed line). Error bars represent SD. Underlying data: S2 Data. **(G)** Ribbon (left) and surface representations (right) of HLA-DR showing the H18 binding surfaces S1 and S2. β-sheet 3, loop 4, and β-sheet 4 (S1) are in violet, and β-sheet 6, loop 7, and β-sheet 7 (S2) in pink. **(H)** Surface representation of the HLA-DR structure highlighting S1 and S2 among the residues that constitute the H18 binding sites predicted by model 1. The bar graph highlights the percentage of these residues (black coloring) among the predicted H18 interacting residues. For statistical analysis, one-way ANOVA followed by Dunnett test was performed. * *P* < 0.05, ** *P* < 0.01, *** *P* < 0.001, **** *P* < 0.0001, ns, not significant. HLA-DR, human leukocyte antigen DR; MHC-II, major histocompatibility complex class II.

Infection of HEK293T cells expressing MHC-II chimeras revealed that swapping the α1, α2, and/or β1 domains of HLA-DR with those of HLA-DM prevented H18-mediated viral entry (Fig 1F), suggesting that each of these domains might contribute to binding H18. To further specify how H18 interacts with MHC-II, we performed in silico docking of the H18 HA1 trimer and the HLA-DR heterodimer. In agreement with our experimental data, 2 models (model 1 and model 2) passed our selection criteria (see Material and methods). Both mapped the H18 binding site to the α1, α2, and β1 domains of HLA-DR (Figs 2A, 2B and S4A Fig) and suggested involvement of 30 (model 2) to 39 (model 1) residues of HLA-DR that buried a total surface area of 1,110 Å^2^ (model 2) to 1,370 Å^2^ (model 1) (Figs 2B and S4A and S1 Table). The models revealed the α2 domain as essential for the interaction with H18, making up between 66.7% (26 of 39 residues in model 1) and 73.3% (22 of 30 residues in model 2) of the putative H18-binding site, with the α1 domain contributing between 16.7% (5 out of 30 residues, model 2) and 25.6% (10 of 39 residues, model 1) and the β1 domain only 3 residues to the interacting surface (Figs 2B and S4A and S1 Table). Accordingly, the α2 domain buried most interface in both models: 940 Å^2^ (model 1) or 740 Å^2^ (model 2), compared to 330 Å^2^ (model 1) or 210 Å^2^ (model 2) contributed by the α1 domain, and 100 Å^2^ (model 1) or 170 Å^2^ (model 2) by the β1 domain (Figs 2B and S4A and S1 Table).

Since the α2 domain makes up the majority of the putative H18 binding surface, we set out to identify the regions critical for viral cell entry within this domain. Based on chimera number 6 (α1, β1, β2 of HLA-DR, α2 of HLA-DM; Fig 1D), we generated MHC-II constructs with chimeric HLA-DR/DM α2 domains, replacing secondary structure motifs (loops and β-sheets) in the α2 domain of HLA-DR with their corresponding HLA-DM counterparts (Fig 2C and 2D). The loops 1 to 3 and β-sheets 1 to 2 form the core of the Ig-like fold and thus are not surface exposed in the HLA-DR crystal structure (S4B Fig). Therefore, we assumed that they should not be involved in H18-mediated entry. Indeed, replacing these regions with the respective HLA-DM α2 sequences (chimera 6a) (Fig 2C) resulted in surface expression (Fig 2E, S2 Data) and viral entry similar to that of wild-type HLA-DR (Figs 2F and S3B, S2 Data). In fact, cell surface expression patterns of all the chimeras were unaffected when we replaced any other individual motif (loops 4 to 7, β-sheets 3 to 7) with the corresponding sequence of HLA-DM (Fig 2E, S2 Data). Yet, only chimeras 6e to 6g supported viral entry, with infection rates of 40% to 70% relative to HLA-DR (Fig 2F, S2 Data), suggesting that loop 5, β-sheet 6, and loop 6 are either not essential for the interaction with H18 or contain critical residues that are also conserved in the corresponding HLA-DM sequences. Chimeras 6b, 6c, 6d, 6h, and 6j failed to mediate VSV-H18 infection (Figs 2F and S3B, S2 Data), suggesting that β-sheet 3, loop 4, β-sheet 4, β-sheet 6, loop 7, and β-sheet 7 of the HLA-DR α2 domain are supporting the interaction with H18. These regions form 2 structural motifs on the surface of HLA-DR, which we refer to as surface 1 (S1, made of β-sheet 3, loop 4, and β-sheet 4) and surface 2 (S2, made of β-sheet 6, loop 7, and β-sheet 7) (Fig 2G). The 19 residues of S1 and S2 combined account for 73% to 86% of the α2 MHC-II residues predicted by the models to interact with H18 (Figs 2H and S4D).

### Conserved amino acids in loop 4 and β-sheet 6 of the HLA-DR α2 domain are critical for H18-mediated entry

**Fig 3:**
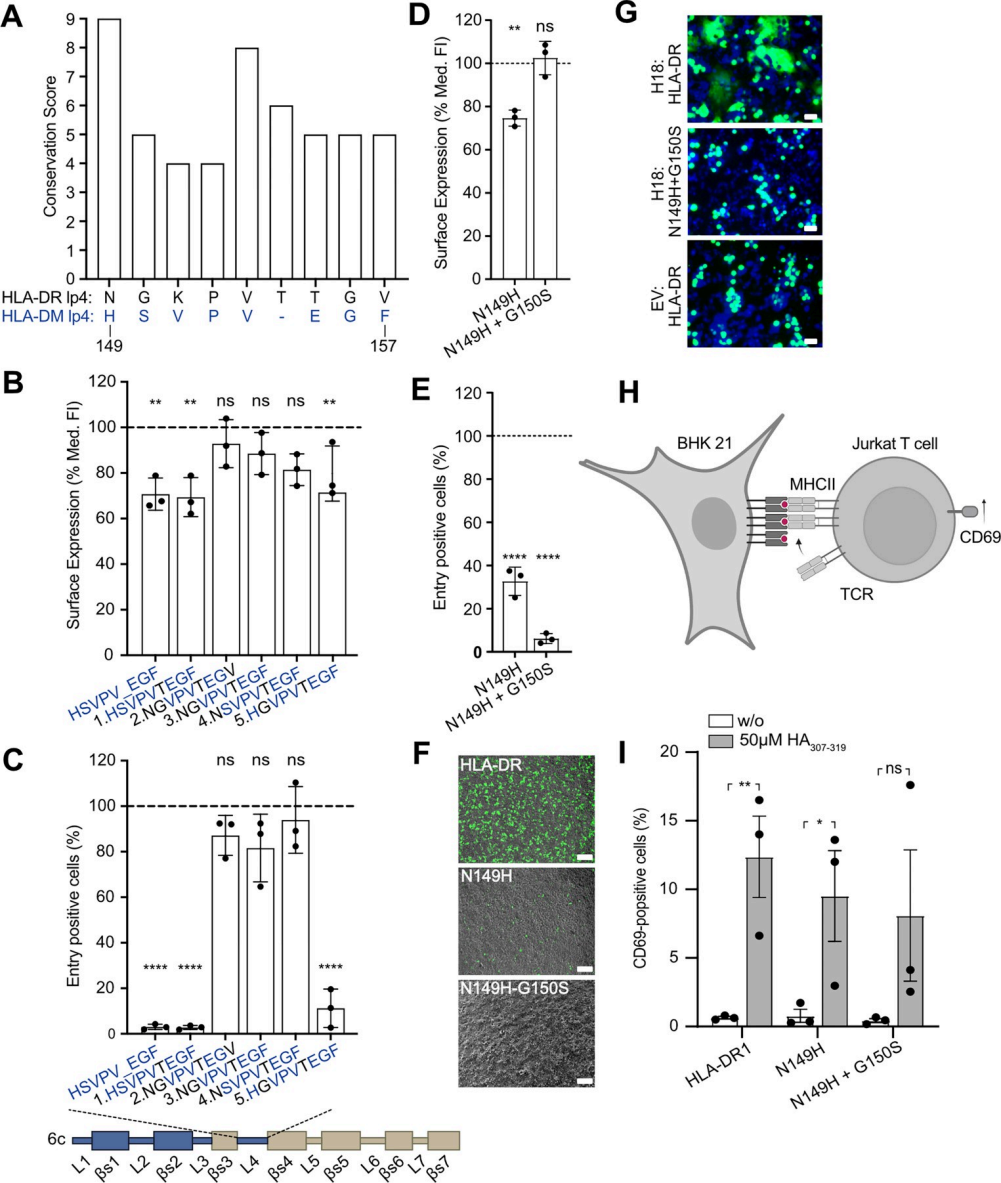
N149 and G150 within the HLA-DR α2 loop 4 are critical for H18-mediated entry. **(A)** Cross-species conservation of the respective amino acids of HLA-DR loop 4 across 300 homologs calculated with ConSurf-DB [21,22]. The bar graph depicts the conservation score for each amino acid ranging from 1 (lowest conservation) to 9 (highest conservation). The corresponding HLA-DM sequence is depicted in blue. Underlying data: S3 Data. **(B)** Surface expression of the variants of the chimera 6c harboring the indicated HLA-DM-specific point mutations in loop 4 on transfected HEK293T cells. The bar graph depicts the median fluorescent intensity of the β-chain determined from cells that fall into the HLA-D α-chain and HLA-D β-chain double positive gate (S1B Fig). Values were normalized to wild-type HLA-DR (dashed line). Underlying data: S3 Data. **(C)** VSV-H18 infection rate of cells expressing the indicated chimera number 6c variants. The percentage of GFP-positive cells is normalized to that of infected HLA-DR-expressing cells. Underlying data: S3 Data. **(D, E)** Surface expression of HLA-DR harboring the indicated HLA-DM-specific amino acid substitutions in loop 4 in transfected HEK293T cells (D) and VSV-H18 infection rate (E). Underlying data: S3 Data. **(F)** GFP-positive cells observed by fluorescent microscopy in HEK293T cells expressing the indicated HLA-DR variants (scale bar, 100 μm). **(G)** pH-induced polykaryon formation of HEK293T cells expressing GFP and H18 or an EV with BHK21 cells expressing the indicated HLA-DR variants. Images are representatives of 3 independent experiments. **(H)** Principle of the T cell activation assay. BHK21 cells transiently expressing HLA-DR1 (HLA-DRB1*01:01) were loaded with the HA_307-319_ peptide and cocultured with CH7C17Jurkat T cells transgenic for the HA1.7 TCR. Binding of the HA1.7 TCR to HLA-DR1–HA_307-319_ peptide complex results in the surface expression of CD69, an early marker of T cell activation. Scheme was created with BioRender.com. **(I)** T cell activation by BHK21 cells transiently expressing HLA-DR1 with the indicated HLA-DM-specific point mutations in the loop 4 of the α2 domain relative to wild-type HLA-DR1. Underlying data: S3 Data. Error bars represent SD. For statistical analysis, one-way ANOVA followed by Dunnett test was performed for panels B-E, and unpaired Student *t* test was used for panel I. * *P* < 0.05, ** *P* < 0.01, **** *P* < 0.0001, ns: not significant. EV, empty vector; HLA-DR, human leukocyte antigen DR; TCR, T cell receptor.

The putative H18-binding site in S1 consists of β-sheet 3, loop 4, and β-sheet 4, with loop 4 forming a prominent kink (Fig 2G, left panel). We hypothesized that this unusual structural feature might contribute to its interaction with H18, and thus we sought to identify the residues within loop 4 required for virus entry. We performed targeted mutagenesis of loop 4 to render a version of HLA-DR/DM-α2 chimera 6c, which would be permissive to H18-mediated infection. While loop 4 of classical MHC-II molecules comprises 9 amino acids, loop 4 of HLA-DM exhibits a deletion of T154 (Fig 3A). We therefore inserted T154 into chimera 6c, designated chimera 6c1, and determined its efficient cell surface expression (Fig 3B, S3 Data), while infection with VSV-H18 failed (Figs 3C and S3C, S3 Data). Starting from chimera 6c1, we then replaced amino acids H149, S150, and F157 of HLA-DM with the HLA-DR residues N149, G150, and V157 to obtain chimera 6c2. This construct was expressed at the cell surface (Fig 3B, S3 Data) and allowed efficient virus entry (Figs 3C and S3C, S3 Data). Subsequent backward mutagenesis of the individual residues N149H, G150S, and V157F, in the chimera 6c2 context, indicated that N149H alone (chimera 6c4) was sufficient to prevent H18-mediated entry (Figs 3C and S3C, S3 Data) revealing a crucial function of N149. In line with this finding, evolutionary conservation analysis using the ConSurf Database [21,22] showed a striking cross-species conservation of this position based on 300 sequence homologs (Conservation score: 9/9) (Fig 3A, S3 Data). To further validate the central role of N149 for H18-mediated entry, we introduced the same HLA-DM-derived substitution N149H into the wild-type HLA-DR (HLA-DR_N149H_). HLA-DR_N149H_ exhibited a slightly lower cell surface expression (Fig 3D, S3 Data), and an infection rate decreased to 35% relative to the wild-type HLA-DR (Fig 3E and 3F, S3 Data). Since chimera 6c5, carrying the S150G substitution, supported minimal virus entry (10%) (Fig 3C), we speculated that G150 was also involved in virus entry to some extent. Indeed, a double substitution of N149H and G150S in wild-type HLA-DR (HLA-DR_N149H+G150S_) resulted in an VSV-H18 infection rate ≤5% (Fig 3E and 3F) despite its efficient surface expression (Fig 3D). To confirm the critical role of N149 and G150 for viral entry, we tested the ability of HLA-DR_N149H+G150S_ to promote cell fusion with H18-expressing cells. While wild-type HLA-DR supported cell fusion with H18-expressing cells and supported polykaryon formation, HLA-DR_N149H+G150S_ failed to mediate cell fusion under the same conditions.

To investigate whether the decreased ability of HLA-DR_N149H_ and HLA-DR_N149H+G150S_ to mediate viral entry is not caused by an overall disruption of the MHC-II structure, we tested their function in a T cell activation assay (Fig 3H). For this purpose, we coexpressed the wild-type HLA-DRA, HLA-DRA_N149H_, or HLA-DRA_N149H+G150S_ with the β chain encoded by the HLA-DRB1 allele 01 (genotype: HLA-DRB1*01:01, serotype: HLA-DR1) in BHK21 cells (S5A–S5D Fig, S5 Data). The MHC-II-expressing BHK21 cells were then loaded with a peptide (HA_307-319_) and cocultured with the CH7C17 Jurkat T cell line expressing the transgenic TCR HA1.7, which specifically recognizes HA_307-319_ loaded on HLA-DR1 (Fig 3H) [23]. As shown in Figs 3I and S5D and S3 and S5 Data files, wild-type HLA-DR and both variants efficiently activated T cells in a peptide-dependent manner as judged by the expression of the T cell activation marker CD69, confirming that neither N149H nor N149H+G150S adversely affected the overall structural integrity of the HLA-DR complex. These results show that the highly conserved amino acids N149 and (to a lesser extent) G150 in loop 4 of HLA-DR α2 are critical for mediating H18-mediated cell entry.

### The architecture of the pocket formed by β-sheet 6, loop 7, and β-sheet 7 of HLA-DR α2 domain is crucial for H18-mediated entry

**Fig 4:**
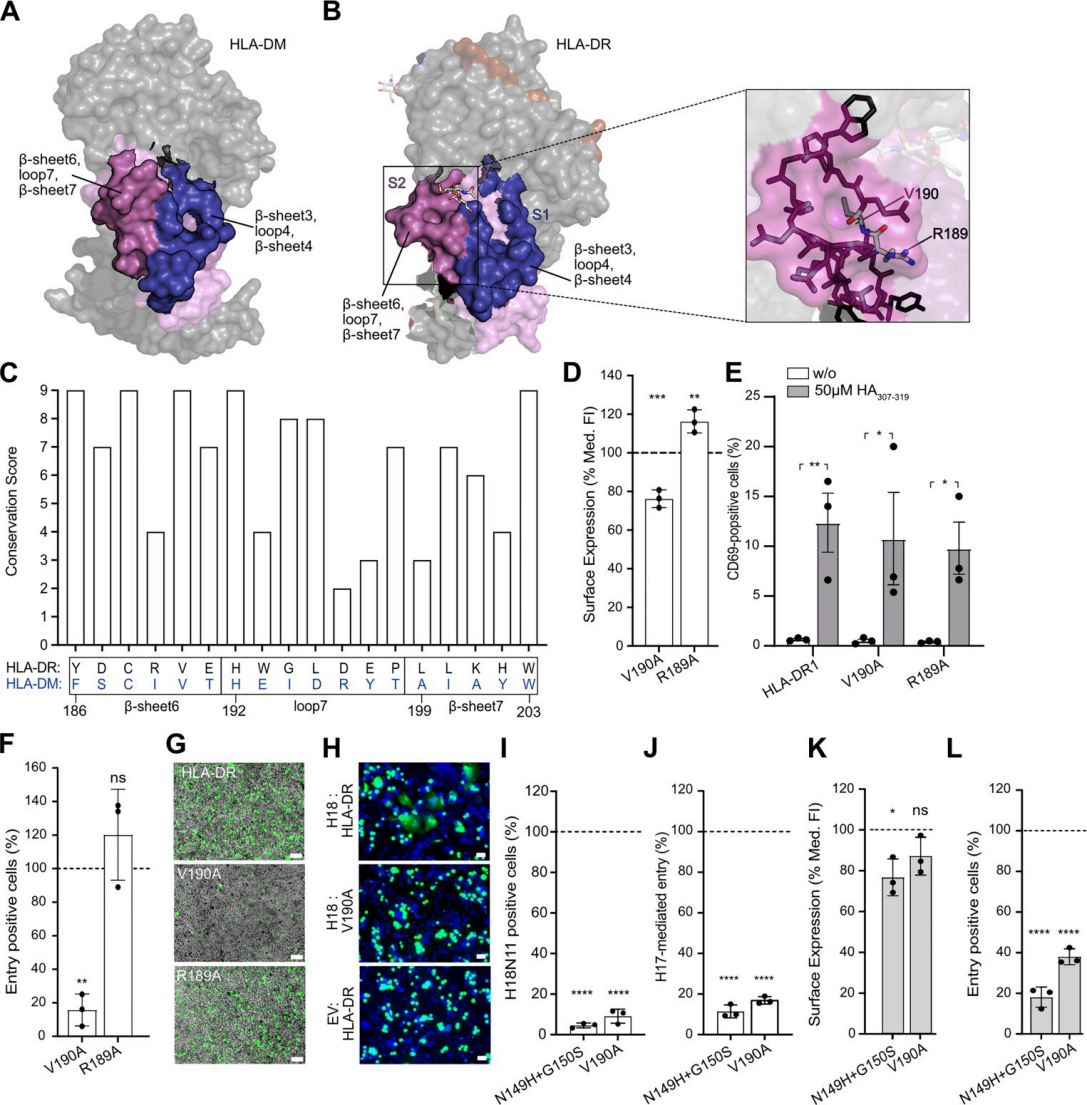
An HLA-DR α2 pocket formed by β-sheet 6, loop 7, and β-sheet 7 is crucial for H18-mediated entry. **(A, B)** Surface representation of HLA-DM (A) and HLA-DR (B, left panel) (PDB codes: 2BC4 and 1DLH, respectively) [41,42] highlighting the H18 binding surfaces S1 and S2. β-sheet 3, loop 4, and β-sheet 4 (S1) are highlighted in violet, while β-sheet 6, loop 7, and β-sheet 7 (S2) are highlighted in pink. Stick representation of the amino acid make-up of the narrow pocket formed by β-sheet 6, loop 6, and β-sheet 7 on HLA-DR (B, right panel), highlighting positions V190 and R189. **(C)** Cross-species conservation of the respective amino acids of HLA-DR β-sheet 6, loop 7, and β-sheet 7 across 300 homologs calculated with ConSurf-DB [21,22]. The bar graph depicts the conservation score for each amino acid ranging from 1 (lowest conservation) to 9 (highest conservation). The corresponded HLA-DM sequence is depicted in blue. Underlying data: S4 Data. **(D)** Flow cytometric analysis of the surface expression of HLA-DR harboring the indicated amino acid substitutions in HEK293T. The bar graph depicts the median fluorescent intensity of the β-chain determined from cells that fall into the HLA-D α-chain and HLA-D β-chain double positive gate (S1B Fig). Values were normalized to wild-type HLA-DR (dashed line). **(E)** T cell activation by BHK21 cells expressing HLA-DR1 with the V190A and R189A mutation relative to wild-type HLA-DR1. Underlying data: S4 Data. **(F, G)** Susceptibility of HEK293T cells expressing the indicated HLA-DR variants to VSV-H18 infection determined by flow cytometry (F) or by fluorescent microscopy (scale bar, 100 μm) (G). Underlying data: S4 Data. **(H)** pH-induced polykaryon formation of HEK293T cells expressing GFP and H18 or EV with BHK21 cells expressing the indicated HLA-DR variants. Images are representatives of 3 independent experiments. **(I, J)** Infection rate of HEK293T cells expressing HLA-DR harboring the indicated mutation with authentic H18N11 virus (I) or VSV-H17 (J). Percentage of entry positive cells are normalized to that of infected, HLA-DR-expressing cells. Underlying data: S4 Data. **(K, L)** Flow cytometric analysis of the surface expression of *Artibeus jamaicensis* MHC-II (Aj-DR) harboring the indicated amino acid substitution in transfected HEK293T cells (K) and their susceptibility to VSV-H18 infection (L). Surface levels are normalized to the wild-type Aj-DR as described in (D). Underlying data: S4 Data. Error bars represent SD. For statistical analysis, one-way ANOVA followed by Dunnett test was performed for panels D, F, I, J, K, and L, and unpaired Student *t* test was used for panel E. * *P* < 0.05, ** *P* < 0.01, *** *P* < 0.001, **** *P* < 0.0001, ns, not significant. EV, empty vector; HEK293T, human embryonic kidney 293T; HLA-DR, human leukocyte antigen DR.

The second putative H18-binding surface (S2) consists of β-sheet 6, loop 7, and β-sheet 7 (Fig 2G). S2 forms a shallow pocket in HLA-DR and a protrusion in HLA-DM (Fig 4A and 4B), prompting us to speculate that the architecture of this region is important for virus entry. The HLA-DR pocket comprises 2 highly conserved hydrophobic amino acids buried at its base: V190 in β-sheet 6 (conservation score: 9/9) and L195 in loop 7 (conservation score: 8/9) (Figs 4C and S4E, S4 Data) To test the importance of structural integrity of the S2 pocket for viral entry, we substituted the highly conserved hydrophobic V190 with an alanine (HLA-DR_**V190A**_) (Fig 4C, S4 Data). As a control, we mutated the poorly conserved R189 (conservation score: 4/9) (Fig 4C, S4 Data) located at the periphery of the pocket (Fig 4B). While both mutants HLA-DR_**V190A**_ and HLA-DR_**R189A**_ were still expressed at the cell surface (Fig 4D, S4 Data) and were able to activate T cells (Fig 4E, S4 Data), HLA-DR_**R189A**_, but not HLA-DR_**V190A**_, allowed for efficient VSV-H18 entry (Fig 4F and 4G, S4 Data). Accordingly, HLA-DR_**V190A**_ was also unable to induce polykaryon formation (Fig 4H), confirming that the pocket formed by β-sheet 6, loop 7, and β-sheet 7 of the α2 domain is also crucial for H18-mediated entry.

To demonstrate the importance of the amino acids N149, G150, and V190 in HLA-DR for authentic H18N11 entry, we infected HEK293T cells transiently expressing mutant HLA-DR_V190A_ and HLA-DR_N149H+G150S_. Indeed, H18N11 entry was barely detectable in cells expressing these mutant HLA-DR complexes (Fig 4I, S4 Data), confirming the importance of the identified residues for cell entry in context of authentic H18N11. Likewise, VSV-H17 entry was greatly diminished in cells expressing HLA-DR_V190A_ and HLA-DR_N149H+G150S_, suggesting that H17 and H18 utilize similar key residues for cell entry (Fig 4J, S4 Data).

### N149, G150, and V190 are also critical for H18-mediated infection in context of MHC-II of the Jamaican fruit bat

We next sought to determine whether residues N149, G150, and V190, which we identified as critical for H18-mediated entry in the context of human HLA-DR, are similarly relevant in the HLA-DR homolog of the Jamaican fruit bat (Aj-DR), a natural reservoir species of H18N11 [24]. Following transient reconstitution, wild-type Aj-DR complex was well expressed at the cell surface of HEK293T cells (Fig 4K, S4 Data) and supported infection of VSV-H18 (Fig 4L, S4 Data). In comparison, Aj-DR harboring the double substitution N149H+G150S (Aj-DR_N149H+G150S_) or the single V190A (Aj-DR_V190A_) mutation reduced the infection rate of VSV-H18 by 80% and 65%, respectively, compared with wild-type Aj-DR (Fig 4L). This suggests that highly conserved amino acids within the MHC-II α2 subunit are required for H18-mediated infection in different species.

### Conserved amino acids in α1 and β1 domains of HLA-DR modulate H18-mediated entry

As shown in Fig 1F, both the α1 and the β1 domains of HLA-DR have a role in H18-mediated entry. Since these domains contribute to the formation of the peptide binding groove almost equally, we tested whether the presence and/or identity of a high-affinity peptide would affect VSV-H18 infection. To this end, we expressed HLA-DR1 molecules having either the HA_307-319_ peptide or the CLIP_87-101_ peptide covalently fused to the N-terminus of the beta chain (HA_307-319_–HLA-DR1 and CLIP_87-101_–HLA-DR1; S6A Fig). Whereas the correct positioning of the fused peptide into the binding groove for HA_307-319_–HLA-DR1 was confirmed by a T cell activation assay (S6B Fig, S6 Data), this was not possible for CLIP_87-101_–HLA-DR1 due to the restriction of the CH7C17 Jurkat T cells to the HA_307-319_ peptide. Both CLIP_87-101_ and HA_307-319_ peptides slightly increased surface expression but substantially improved viral entry (S6C and S6D Fig, S6 Data). We speculate that covalent fusion of a high-affinity peptide stabilizes the MHC-II molecule and thereby facilitates viral entry in a sequence-independent manner. Indeed, binding of a peptide to an empty MHC-II was shown to increase stability at both neutral and acidic pH [25].

To further determine the importance of highly conserved amino acids within the α1 and the β1 domains of HLA-DR for viral entry, individual amino acids therein were substituted with their HLA-DM counterparts or alanine when identical (S7A Fig). Only surface-exposed amino acids that were present in 75% of all MHC-II homologs and also had a conservation score >8 (ConSurf-DB Score) were considered. All the corresponding MHC-II mutants were surface expressed at levels comparable to wild-type HLA-DR (S7B Fig, S7 Data). Viral entry of VSV-H18 was only substantially impaired (>30% reduction of entry positive cells) in cells expressing the MHC-II mutants M61F (α1 domain) or N91A (β1 domain) (S7C Fig, S7 Data). Importantly, M61 of the α1 domain forms part of the predicted H18-binding site, which further supports our in silico modeling approach (S7D Fig, S1 Table).

## Discussion

We elucidated the central role of the α2 domain of the MHC-II molecule in H18-mediated cell entry by generating sets of chimeras between the classical human MHC-II, HLA-DR, and the nonclassical MHC-II molecule, HLA-DM. Within the α2 domain, we identified the highly conserved amino acid residues N149 and V190 as critical for both H18- and H17-mediated infection. Single substitutions of these amino acids in HLA-DR prevented viral entry but did not affect the structural integrity of the HLA-DR molecules as these mutants were still able to accommodate antigenic peptides and activate T cells. The fact that these HLA-DR mutants were able to activate T cells demonstrates their overall structural integrity and clustering ability. The latter, which is critical for T cell activation, may also provide the avidity required for virus attachment and entry [19,26,27]. The α2 and β2 domains of MHC-II molecules are C1-set Ig-like domains [28], which makes them well suited to act as platforms for interactions with other proteins [29]. Indeed, the α2 domain, which we found to be crucial for H18N11 entry, was previously shown to bind TIRC7, a negative regulator of T cell activity [30]. Furthermore, the HLA-DR:CD4 and HLA-DR:HLA-DM interactions consist of large multidomain interfaces in which the α2 domain is a major contributor [31–33]. Our in silico structural modeling suggests that the interaction with H18 occurs through an interface involving the α2 domain and parts of the α1 and β1 domains in HLA-DR. Despite the involvement of multiple domains, we hypothesize that the H18:HLA-DR interaction is of relatively low affinity as classical biochemical approaches have yet failed to detect direct binding [11]. However, according to our predicted model, 1 H18 homotrimer can bind to 3 MHC-II complexes (S4F and S4G Fig), which would allow clustering of MHC-II at the viral entry site and provide the avidity required for host cell binding and subsequent uptake. Similar clustering of entry factors is also required for the uptake of classical IAV due to the low affinity of individual HA–sialic acid interactions [34,35].

On a genetic level, the MHC-II α- and β-chains are markedly conserved among all mammalian species with the obvious exception of the polymorphic residues that mainly cluster in the β1 domain [16]. Usage of such a conserved receptor allows bat IAV to infect a wide range of New World bat species, including the phylogenetically only distantly related Neotropical fruit bats, *Artibeus* spp. (family Phyllostomidae), and Velvety free-tailed bat, *Molossus molossus* (family Molossidae) [8]. However, despite the ability to utilize MHC-II of diverse mammalian species and the wide geographical distribution of seropositive bats across Central and South America, there is as of yet no evidence for natural infection of non-bat species with bat IAVs. This might suggest that there are additional molecular and/or ecological hurdles, which so far have prevented a spill over to other mammals including humans.

## Material and methods

### Cell lines

HEK293T cells were obtained from the American Type Culture Collection (ATCC; CRL-3216). Baby Hamster Kidney Fibroblasts (BHK-21) cells were obtained from the German Cell Culture Collection (DSZM). MDCKII cells stably overexpressing human MHC-II (MDCK-MHC-II) were generated previously [11] and selected using 2.5 μg per ml puromycin and 300 μg per ml hygromycin. All cells were cultured in Dulbecco’s Modified Eagle’s Medium (DMEM; Gibco, Thermo Fischer Scientific) containing 10% fetal calf serum (FCS), 100 U per ml penicillin, and 100 mg per ml streptomycin at 37°C with 5% CO_2_.

### Viruses

GFP-encoding vesicular stomatitis virus whose glycoprotein was replaced by H18 with a polybasic cleavage site (VSV-H18) was generated as previously described [20]. Cell culture-adapted H18N11 (rP11) was produced in MDCKII cells stably expressing HLA-DR as previously described [24].

### MHC-II expression plasmids

cDNA sequences encoding V5- or HA-tagged wild-type, and chimeric MHC-II α- and β-chains were synthesized (Genewiz) and cloned into the pCAGGS vector via NotI and XhoI restriction enzymes (S1A Fig). Reference encoding sequences used include: HLA-DRA (NM_019111.4), HLA-DRB1 (NM_001243965.1), HLA-DRB1*0101 (HM067843.1), HLA-DMA (NM_006120.4), HLA-DMB (NM_002118.5), Aj-DRA (XM_037146055.1), and Aj-DRB (XM_037162922.1). Mutations were introduced by PCR using overlapping primers (Sigma Aldrich). Truncated MHC-II β-chain was generated by introduction of the amber stop codon at respective amino acid positions. Amino acid differences in the signal peptide of V5- and HA-tagged wild-type HLA-DR are due to codon optimization. Plasmids encoding HLA-DRB1*01:01 with CLIP_87-101_ (PVSKMRMATPLLMQA) or HA_307-319_ (PKYVKQNTLKLAT) were generated by PCR using overlapping primers (Sigma Aldrich) and cloned into the pCAGGS vector via NotI and XhoI restriction enzymes.

### MHC-II surface expression

HEK293T cells seeded to approximately 70% confluency in 24-well plates were transfected with 250 ng each of the respective MHC-II α- and β-chain using Lipofectamine 2000 (Thermo Fisher, Germany). The next day, cells were detached and washed by pipetting up and down gently with FACS buffer (PBS supplemented with 2% FCS). After centrifugation at 1,200 rpm for 5 min at 4°C, cells were stained primarily with anti-V5 rabbit antibody (Abcam, catalog no. ab9116, 1:500) and anti-HA mouse antibody (Sigma Aldrich, catalog no. H3663, 1:500) for 30 min on ice. Following another washing and centrifugation step, cells were secondarily stained with BV421 goat anti-rabbit antibody (BD Biosciences, catalog no. 565014, 1:200) and APC goat anti-mouse antibody (BD Biosciences, catalog no. 550826, 1:200) for 30 min on ice. Zombie NIR Fixable Viability Kit (BioLegend, catalog no. 423105, 1:1000) was used to assess live versus dead status of cells. After a final wash and centrifugation step, cells were resuspended in FACS buffer, transferred to a FACS tube, and surface expression of MHC-II heterodimer was analyzed with a BD FACS Canto II (BD Biosciences) flow cytometer.

### Virus infections

HEK293T cells seeded to approximately 70% confluency in 24-well plates were transfected with 250 ng each of the respective MHC-II α- and β-chain using Lipofectamine 2000 (Thermo Fisher, Germany). For VSV-H18 infection, cells were infected 24 h posttransfection at an MOI of 0.05 in infection medium [11]. At 24 h postinfection, cells were detached, washed, centrifuged, and stained as described for MHC-II surface expression. After staining and washing, cells were fixed in 2% PFA in PBS for 20 min on ice, washed, and centrifuged at 1,500 rpm for 10 min at 4°C. After a final wash and centrifugation step, cells were resuspended in FACS buffer, transferred to a FACS tube, and MHCII surface expression as well as the frequency of infected GFP-positive cells were analyzed with a BD FACS Canto II (BD Biosciences) or BD LSRFortessa (BD Biosciences) flow cytometer.

For H18N11 infection, cells were infected 24 h posttransfection at an MOI of 5 in infection medium supplemented with 0.2 μg/ml TPCK trypsin. At 24 h postinfection, cells were resuspended in infection medium, washed, and centrifuged as described for MHC-II surface expression. Subsequently, cells were primarily stained with a rabbit polyclonal anti-H18 serum (1:100) [11] for 30 min on ice. After washing in FACS buffer and centrifugation at 1,200 rpm for 5 min at 4°C, cells were stained with BV421 goat anti-rabbit antibody (BD Biosciences, catalog no. 565014, 1:200) and Alexa Fluor 488–conjugated HA tag monoclonal antibody (Thermo Fisher, catalog no. A-21287, 1:200) for 30 min on ice. After washing and centrifugation, cells were fixed in 2% PFA in PBS for 20 min on ice, washed, and centrifuged at 1,500 rpm for 10 min at 4°C. After a final wash step, cells were resuspended in FACS buffer, transferred to a FACS tube, and MHC-II surface expression as well as the frequency of H18N11-infected Alexa Fluor 488–positive cells were analyzed with BD LSRFortessa (BD Biosciences) flow cytometer.

Fluorescent images of GFP-positive VSV-H18-infected cells were acquired on a Zeiss Observer.ZI inverted epifluorescence microscope (Carl Zeiss) equipped with an AxioCamMR3 camera using a 10× objective.

### Molecular docking

The HDOCK server (http://hdock.phys.hust.edu.cn/) [36–38] was used to computationally construct the three-dimensional (3D) complex model of H18:MHC-II using crystal structures of MHC-II molecules and H18 HA deposited in the PDB and its default hybrid docking protocol. Focusing on the HA1 region that binds the sialic acid receptor of conventional IAVs [39,40] and acquired amino acid mutations that increase replication competence of H18N11 [24], the PDB entry 4K3X [8], (chains A, C, E) of H18 were designated as the “interactor” and MHCII (PDB ID 1DLH, chains A, B) [41] as the “ligand.” After a global sampling of putative binding orientations at 15° rotational intervals, HDOCK provided docking results, including 10 top models based on its scoring function. Among these top 10 docking models, the top 2 models with the lowest docking score (below −200; the most possible binding model) and confidence score greater than 0.7 (very likely to bind), in addition to the criteria of using the HA head domain, not engaging the HLA-DR peptide binding groove, and being in upright orientation, were selected. Pymol was used for 3D structure visualization of H18:MHC-II complex model and PISA was used to calculate buried surface areas.

### T cell activation

Baby Hamster Kidney Fibroblasts (BHK-21) (8.4 × 10^5^ cells per well in 6-well format) were transfected with 2 pCAGGS expression vectors (500 ng each) encoding the HLA-DRB1*01:01 β-chain and the respective HLA-DRA1 α-chain using Lipofectamine 2000 (Thermo Fisher, Germany). The next day, 5 × 10^4^ transfected cells were transferred into a well of a 96-well plate and cultured over night at 37°C in DMEM (Gibco, USA) containing 1% FCS. For exogenous peptide loading, 50 μM HA_307-319_ peptide (PKYVKQNTLKLAT; GenScript, USA) or EBV EBNA1_515-527_ peptide (TSLYNLRRGTALA; GENAXXON bioscience, Germany) were added to the culture medium. Medium was removed and cells were cocultured with CH7C17 Jurkat T cells (10^5^ cells per well in 96-well format) in RPMI 1640 (Gibco, USA) supplemented with 10% FCS and 5% HEPES for 6 h at 37°C. Subsequently, cells were stained with FITC-labeled anti-CD3 antibody (BioLegend, USA, 1:200) and APC-labeled anti-CD69 antibody (Life Technologies, USA, 1:200) and analyzed with a BD FACSCanto II (BD Biosciences) flow cytometer.

### Polykaryon formation assay

Subconfluent HEK293T cells were cultured in 6-well plates and cotransfected with 2 μg of pCAGGS-GFP and either pCAGGS-EV (empty vector) or pCAGGS-H18. Similarly, subconfluent BHK-21 cells were cotransfected with 2 μg of pCAGGS-HLA-DRB1 and either pCAGGS-HLA-DRA or pCAGGS-HLA-DRA_N149H+G150S_ or pCAGGS-HLA-DRA_V190A_. At 24 h posttransfection, cells were detached by trypsin treatment and 2 × 10^5^ HEK293T and 2 × 10^5^ BHK21 cells, respectively, were seeded in collagen coated 24-well plates containing growth medium (DMEM, 10% FCS, 100 U per ml penicillin, and 100 mg per ml streptomycin) and incubated at 37°C and 5% CO_2_. The following day, cells were treated with TPCK trypsin (10 μg/ml in Opti-MEM) for 30 min at 37°C. Cells were subsequently washed with PBS, exposed to pH 5 PBS for 20 min at 37°C and 5% CO_2_, and then incubated in growth medium for 2 h at 37°C and 5% CO_2_. Finally, cells were washed with PBS, fixed using 4% paraformaldehyde in PBS for 20 min, and nuclei were stained for 1 h using 4′,6-diamidino-2-phenylindole (DAPI). Fluorescence images were acquired using a Zeiss Observer.ZI inverted epifluorescence microscope (Carl Zeiss) equipped with an AxioCamMR3 camera using a 20× objective.

### Conservation analysis

To determine the cross-species conservation of amino acids within the α- and β-chain of HLA-DR, we performed a ConSurf-DB analysis [21,22] for 1DLH chain A and B (PDB DOI: 10.2210/pdb1DLH/pdb) [41]. The calculation was conducted on 300 hits out of 1,872 (α-chain) and 5,558 (β-chain) homologs, which were CT-HIT unique at 95% threshold. Where necessary, resulting conservation scores were plotted for our regions of interest using GraphPad Prism.

## Supporting information

S1 Table H18-binding residues predicted by model 1 and model 2 within the indicated HLA-DR domains. (TIFF)

S1 Fig Amino acid sequence alignment of HLA-DR and HLA-DMtc. **(A, B)** Amino acid sequence alignment of HLA-DRA and HLA-DMAtc (A) and HLA-DRB1 and HLA-DMBtc (B), highlighting the features of each construct and the respective domains used for chimeric MHC-II generation. Identical residues are highlighted in black boxes and similar residues in gray boxes. HLA-DR, human leukocyte antigen DR; MHC-II, major histocompatibility complex class II.
(TIFF)

S2 Fig Flow cytometric gating strategy for MHC-II surface expression, virus entry, and T cell activation. **(A)** Schematic representation of the DNA construct design used for the expression of V5-tagged MHC-II α- and HA-tagged MHC-II β-chains. **(B-D)** Representative flow cytometric plots showing the gating strategy applied to determine MHC-II heterodimer surface expression (B) as well as VSV-H18 and VSV-H17 (C) and H18N11 (D) infection rates. SSC-A vs. FSC-A gating was performed to identify cells of interest. FSC-H vs. FSC-A gating was used for doublet exclusion. Cells staining positive for the amine reactive cell viability dye (Zombie-NIR) were considered dead (B). **(E)** Representative flow cytometric plots showing the gating strategy used to quantify T cell activation. FSC-A vs. PE-A gating was performed to exclude autofluorescent cells. FSC, forward scatter; MHC-II, major histocompatibility complex class II; PE, phycoerythrin; SSC, side scatter.
(TIFF)

S3 Fig Fluorescent microscopic images of VSV-H18 infection. **(A-C)** Susceptibility of HEK293T cells transfected with the respective MHC-II complexes to infection with a GFP-encoding vesicular stomatitis virus comprising H18 in place of the VSV-Glycoprotein (VSV-H18). Scale bars represent 100 μm. Images are representatives of 3 independent experiments. EV, empty vector; HEK293T, human embryonic kidney 293T; MHC-II, major histocompatibility complex class II.
(TIFF)

S4 Fig Structural representations of HLA-DR, HLA-DM, and H18:HLA-DR. **(A)** Surface representation of the crystal structure of HLA-DR bound to a peptide highlighting the H18 binding sites predicted by model 2 (shown in wine color) (left). The bar graph depicts the number of HLA-DR residues that contribute to H18 binding within the indicated domains and in total. Numbers above the graph represent the respective buried surface area involved in H18 binding (right). **(B, C)** Ribbon representation of the crystal structures of HLA-DR (B) and HLA-DM (C), highlighting the loops (teal) and β-sheets (light brown) in the α2 domain. Loops and β-sheets are both numbered 1 to 7. **(D)** Surface representation of HLA-DR, highlighting S1 and S2 within the H18 binding site predicted by model 2 (left), and a bar graph depicting this in percentages, with S1 and S2 residues highlighted in black (right). **(E)** Surface representation of the crystal structure of HLA-DR (PDB code:1DLH) [41], highlighting the H18 binding surfaces (S1 and S2) in the α2 domain of MHC-II (left) and stick representation of the residues of S2 highlighting the highly conserved basic amino acids at the base of the pocket (right). **(F)** Surface representation of the H18:MHC-II complex structure predicted by model 1, showing H18 trimer (HA1) in different shades of blue and MHC-II heterodimer domains in different shades of gray. The putative H18 binding site is highlighted in purple and the putative RBS in yellow. **(G)** View from the perspective of the host membrane of the H18:MHC-II complex predicted by model 1. Here, an H18 trimer interacts with 3 MHC-II heterodimers, both colored as in (F). HLA-DR, human leukocyte antigen DR; MHC-II, major histocompatibility complex class II; RBS, receptor binding site.
(TIFF)

S5 Fig Surface expression, susceptibility, and T cell activation capacity of HLA-DR1. **(A, B)** Flow cytometric analysis of the surface expression of HEK293T cells transfected with HLA-DR1 (HLA-DRB1*01:01) in comparison to HLA-DR15 (HLA-DRB1*15:01) (A) and alone (B). The bar graph depicts the median fluorescent intensity of the β-chain determined from cells that fall into the HLA-DR α-chain and HLA-DR β-chain double positive gate (S1B Fig) and shows similar expression irrespective of the β-chain used. Values were normalized to WT HLA-DR15 (A). The number within the flow cytometry plot indicates the percentage of cells falling into the gate (B). Underlying data: S5 Data. **(C)** Susceptibility of HEK293T cells transfected with HLA-DR1 to VSV-H18. Infected GFP-positive cells among the MHC-II-expressing population (B, left) population were quantified by flow cytometry (C, right) and shown by fluorescent microscopy (scale bar, 100 μm) (C, left). **(D)** T cell activation by BHK21 cells transiently expressing HLA-DR1 loaded with the HA_307-319_ and the EBV EBNA1_515-527_ peptide, respectively. Underlying data: S5 Data. For statistical analysis, unpaired Student *t* test was performed for panel D. * *P* < 0.05. HEK293T, human embryonic kidney 293T; HLA-DR, human leukocyte antigen DR; MHC-II, major histocompatibility complex class II; WT, wild-type.
(TIFF)

S6 Fig T cell activation capacity, surface expression, and susceptibility of HLA-DR1 comprising covalently fused peptides. **(A)** Schematic representation of the DNA construct design used for the expression of MHC-II β-chains (HLA-DRB1*01:01) having the HA_307-319_ peptide or the CLIP_87-101_ peptide covalently fused to the N-terminus. **(B)** T cell activation by BHK21 cells transiently expressing the indicated HLA-DR1 complexes. Underlying data: S6 Data. **(C)** Flow cytometric analysis of surface expression of the indicated HLA-DR1 complexes. Data were normalized to the surface levels of HLA-DR1 without a covalently fused peptide (dashed line). Underlying data: S6 Data. **(D)** Susceptibility of cells expressing the indicated HLA-DR1 complexes to VSV-H18 infection. Data were normalized to the susceptibility of cells expressing HLA-DR1 without a covalently fused peptide (dashed line). Underlying data: S6 Data. For statistical analysis, unpaired Student *t* test was performed for panel B, and one-way ANOVA followed by Dunnett test was used for panels C and D. * *P* < 0.05, ** *P* < 0.01, *** *P* < 0.001, ns, not significant. MHC-II, major histocompatibility complex class II.
(TIFF)

S7 Fig Conserved amino acids in α1 and β1 domains of HLA-DR modulate H18-mediated entry. **(A)** Localization of surface-exposed amino acids in the α1 (gray) and β1 (black) domains of HLA-DR that are present in 75% of all MHC-II homologs and have a conservation score >8. These amino acids were substituted with their HLA-DM counterparts or alanine when conserved. **(B)** Surface expression of the indicated HLA-DR variants on transfected HEK293T cells. The bar graph depicts the median fluorescent intensity of the β-chain determined from cells that fall into the HLA-DR α-chain and HLA-DR β-chain double positive gate. Values were normalized to wild-type HLA-DR (dashed line). Underlying data: S7 Data. **(C)** VSV-H18 infection rate of cells expressing the indicated HLA-DR variants depicted in (B). Underlying data: S7 Data. **(D)** Localization of the highly conserved amino acids substituted with their HLA-DM counterparts or alanine shown in (A) in relation to the predicted H18 binding of model 1 (see also Fig 2H). M61 and D60 are part of the modeled H18 binding surface. For statistical analysis, one-way ANOVA followed by Dunnett test was performed for panels B and C. ns, not significant. HEK293T, human embryonic kidney 293T; HLA-DR, human leukocyte antigen DR; MHC-II, major histocompatibility complex class II.
(TIFF)

S1 Data Data underlying Fig 1B, 1E and 1F. (XLSX)

S2 Data Data underlying Fig 2E and 2F. (XLSX)

S3 Data Data underlying Fig 3A–3E and 3I. (XLSX)

S4 Data Data underlying Fig 4K and 4L. (XLSX)

S5 Data Data underlying S5A and S5D Fig. (XLSX)

S6 Data Data underlying S6B–S6D Fig. (XLSX)

S7 Data Data underlying S7B and S7C Fig. (XLSX)

## Abbreviations

DMEM: Dulbecco’s Modified Eagle’s Medium
FCS: fetal calf serum
HEK293T: human embryonic kidney 293T
HLA-DR: human leukocyte antigen DR
IAV: influenza A virus
Ig: immunoglobulin
MHC-II: major histocompatibility complex class II
