# Supplementary figures and images for: Evolutionarily conserved amino acids in MHC-II mediate bat influenza A virus entry into human cells
Source: PLoS Biol. 2023 Jul 6;21(7):e3002182. doi: 10.1371/journal.pbio.3002182 (PMC10325068; doi:10.1371/journal.pbio.3002182)

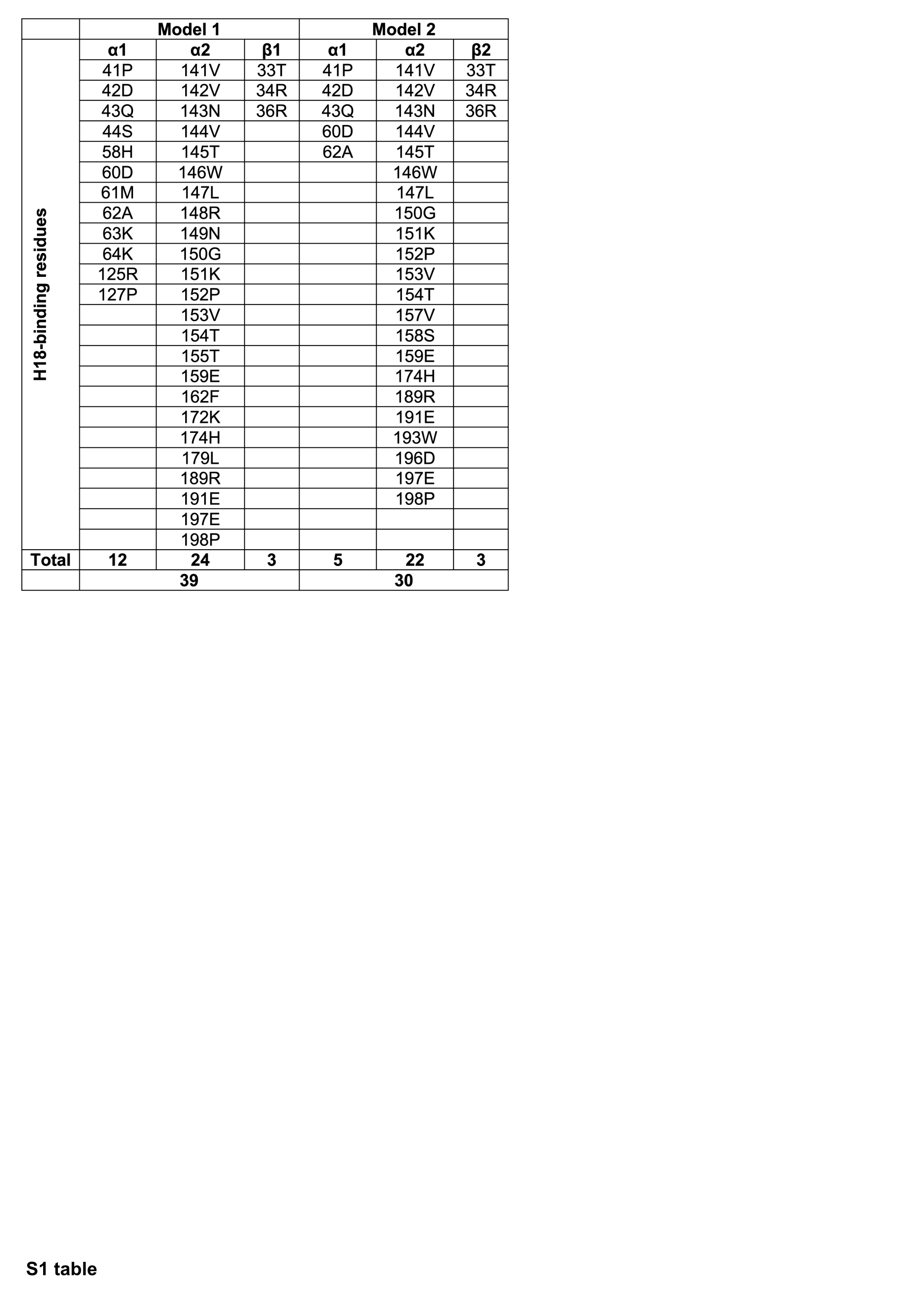

Supplement: S1 Table — (TIFF) [file pbio.3002182.s001.tiff]

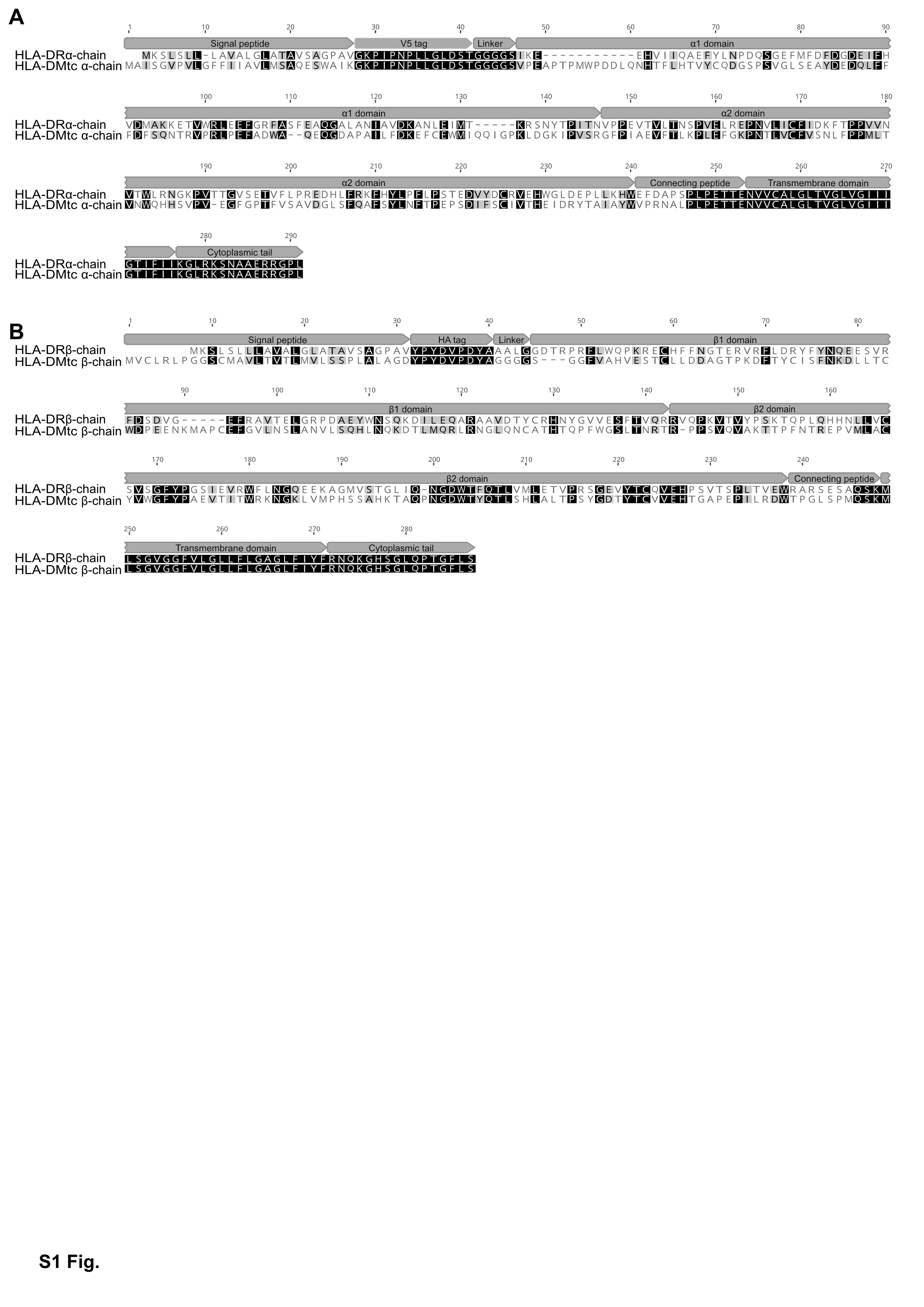

Supplement: S1 Fig — (A, B) Amino acid sequence alignment of HLA-DRA and HLA-DMAtc (A) and HLA-DRB1 and HLA-DMBtc (B), highlighting the features of each construct and the respective domains used for chimeric MHC-II generation. Identical residues are highlighted in black boxes and similar residues in gray boxes. HLA-DR, human leukocyte antigen DR; MHC-II, major histocompatibility complex class II. (TIFF) [file pbio.3002182.s002.tiff]

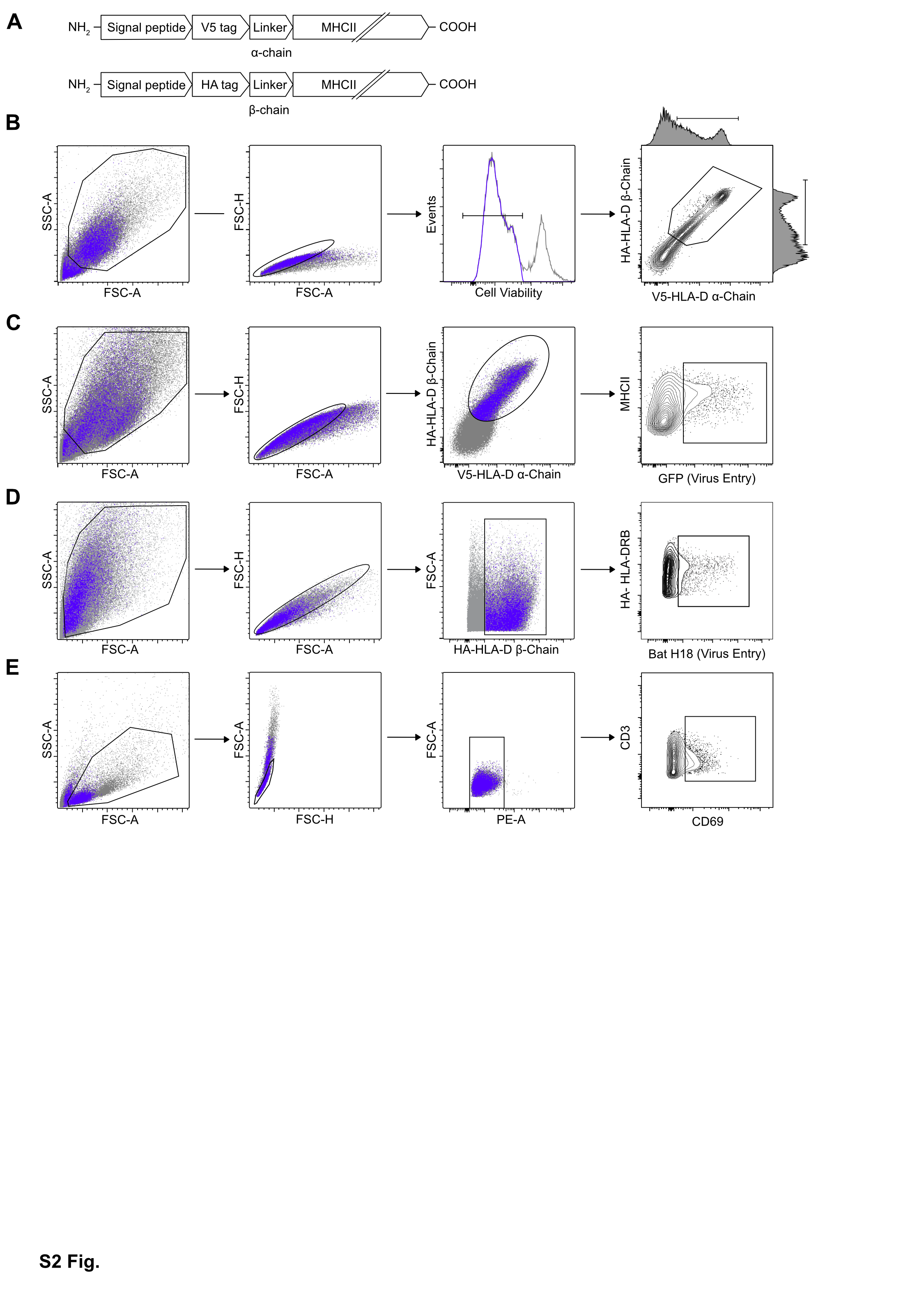

Supplement: S2 Fig — (A) Schematic representation of the DNA construct design used for the expression of V5-tagged MHC-II α- and HA-tagged MHC-II β-chains. (B-D) Representative flow cytometric plots showing the gating strategy applied to determine MHC-II heterodimer surface expression (B) as well as VSV-H18 and VSV-H17 (C) and H18N11 (D) infection rates. SSC-A vs. FSC-A gating was performed to identify cells of interest. FSC-H vs. FSC-A gating was used for doublet exclusion. Cells staining positive for the amine reactive cell viability dye (Zombie-NIR) were considered dead (B). (E) Representative flow cytometric plots showing the gating strategy used to quantify T cell activation. FSC-A vs. PE-A gating was performed to exclude autofluorescent cells. FSC, forward scatter; MHC-II, major histocompatibility complex class II; PE, phycoerythrin; SSC, side scatter. (TIFF) [file pbio.3002182.s003.tiff]

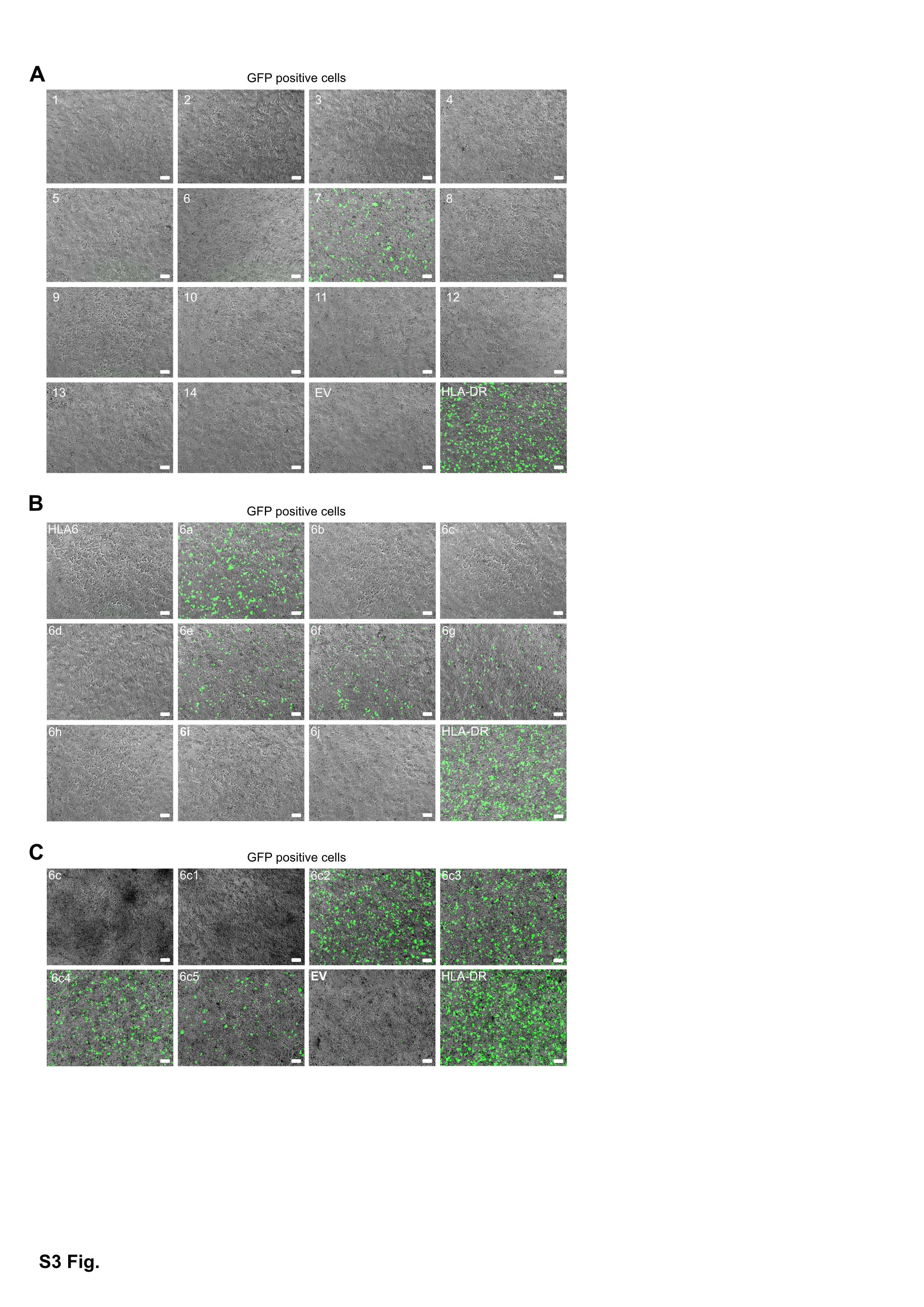

Supplement: S3 Fig — (A-C) Susceptibility of HEK293T cells transfected with the respective MHC-II complexes to infection with a GFP-encoding vesicular stomatitis virus comprising H18 in place of the VSV-Glycoprotein (VSV-H18). Scale bars represent 100 μm. Images are representatives of 3 independent experiments. EV, empty vector; HEK293T, human embryonic kidney 293T; MHC-II, major histocompatibility complex class II. (TIFF) [file pbio.3002182.s004.tiff]

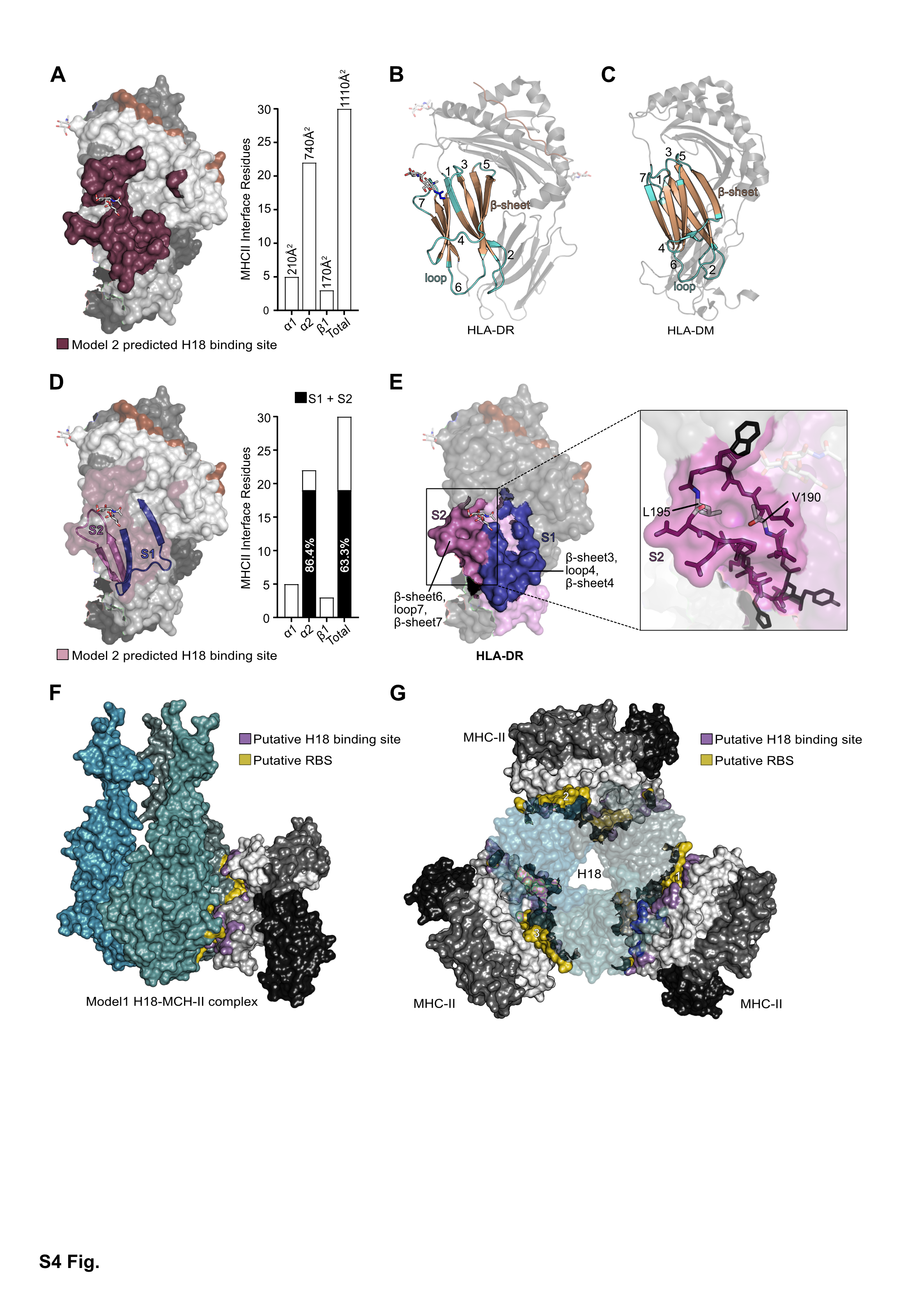

Supplement: S4 Fig — (A) Surface representation of the crystal structure of HLA-DR bound to a peptide highlighting the H18 binding sites predicted by model 2 (shown in wine color) (left). The bar graph depicts the number of HLA-DR residues that contribute to H18 binding within the indicated domains and in total. Numbers above the graph represent the respective buried surface area involved in H18 binding (right). (B, C) Ribbon representation of the crystal structures of HLA-DR (B) and HLA-DM (C), highlighting the loops (teal) and β-sheets (light brown) in the α2 domain. Loops and β-sheets are both numbered 1 to 7. (D) Surface representation of HLA-DR, highlighting S1 and S2 within the H18 binding site predicted by model 2 (left), and a bar graph depicting this in percentages, with S1 and S2 residues highlighted in black (right). (E) Surface representation of the crystal structure of HLA-DR (PDB code:1DLH) [41], highlighting the H18 binding surfaces (S1 and S2) in the α2 domain of MHC-II (left) and stick representation of the residues of S2 highlighting the highly conserved basic amino acids at the base of the pocket (right). (F) Surface representation of the H18:MHC-II complex structure predicted by model 1, showing H18 trimer (HA1) in different shades of blue and MHC-II heterodimer domains in different shades of gray. The putative H18 binding site is highlighted in purple and the putative RBS in yellow. (G) View from the perspective of the host membrane of the H18:MHC-II complex predicted by model 1. Here, an H18 trimer interacts with 3 MHC-II heterodimers, both colored as in (F). HLA-DR, human leukocyte antigen DR; MHC-II, major histocompatibility complex class II; RBS, receptor binding site. (TIFF) [file pbio.3002182.s005.tiff]

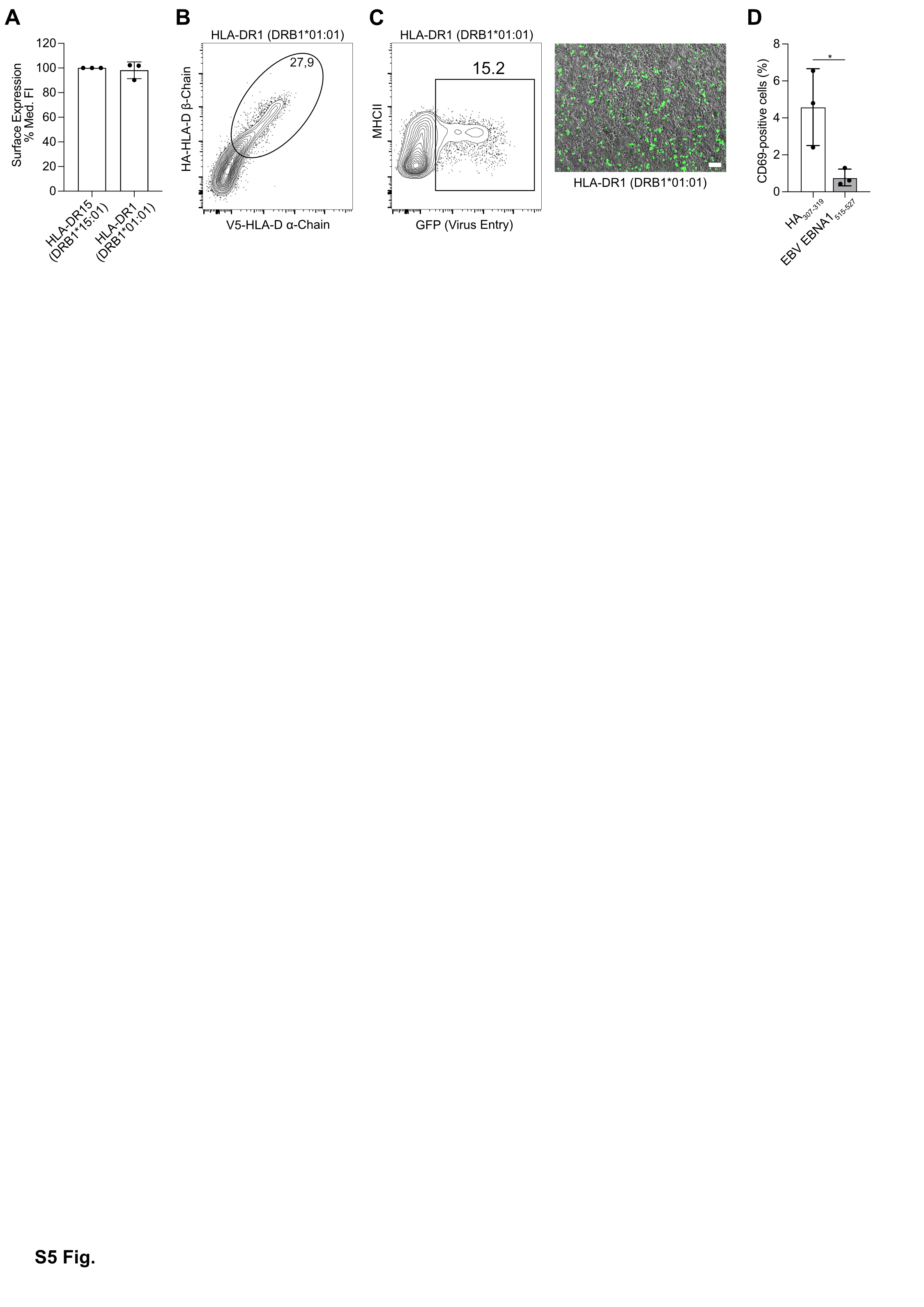

Supplement: S5 Fig — (A, B) Flow cytometric analysis of the surface expression of HEK293T cells transfected with HLA-DR1 (HLA-DRB1*01:01) in comparison to HLA-DR15 (HLA-DRB1*15:01) (A) and alone (B). The bar graph depicts the median fluorescent intensity of the β-chain determined from cells that fall into the HLA-DR α-chain and HLA-DR β-chain double positive gate (S1B Fig) and shows similar expression irrespective of the β-chain used. Values were normalized to WT HLA-DR15 (A). The number within the flow cytometry plot indicates the percentage of cells falling into the gate (B). Underlying data: S5 Data. (C) Susceptibility of HEK293T cells transfected with HLA-DR1 to VSV-H18. Infected GFP-positive cells among the MHC-II-expressing population (B, left) population were quantified by flow cytometry (C, right) and shown by fluorescent microscopy (scale bar, 100 μm) (C, left). (D) T cell activation by BHK21 cells transiently expressing HLA-DR1 loaded with the HA307-319 and the EBV EBNA1515-527 peptide, respectively. Underlying data: S5 Data. For statistical analysis, unpaired Student t test was performed for panel D. * P < 0.05. HEK293T, human embryonic kidney 293T; HLA-DR, human leukocyte antigen DR; MHC-II, major histocompatibility complex class II; WT, wild-type. (TIFF) [file pbio.3002182.s006.tiff]

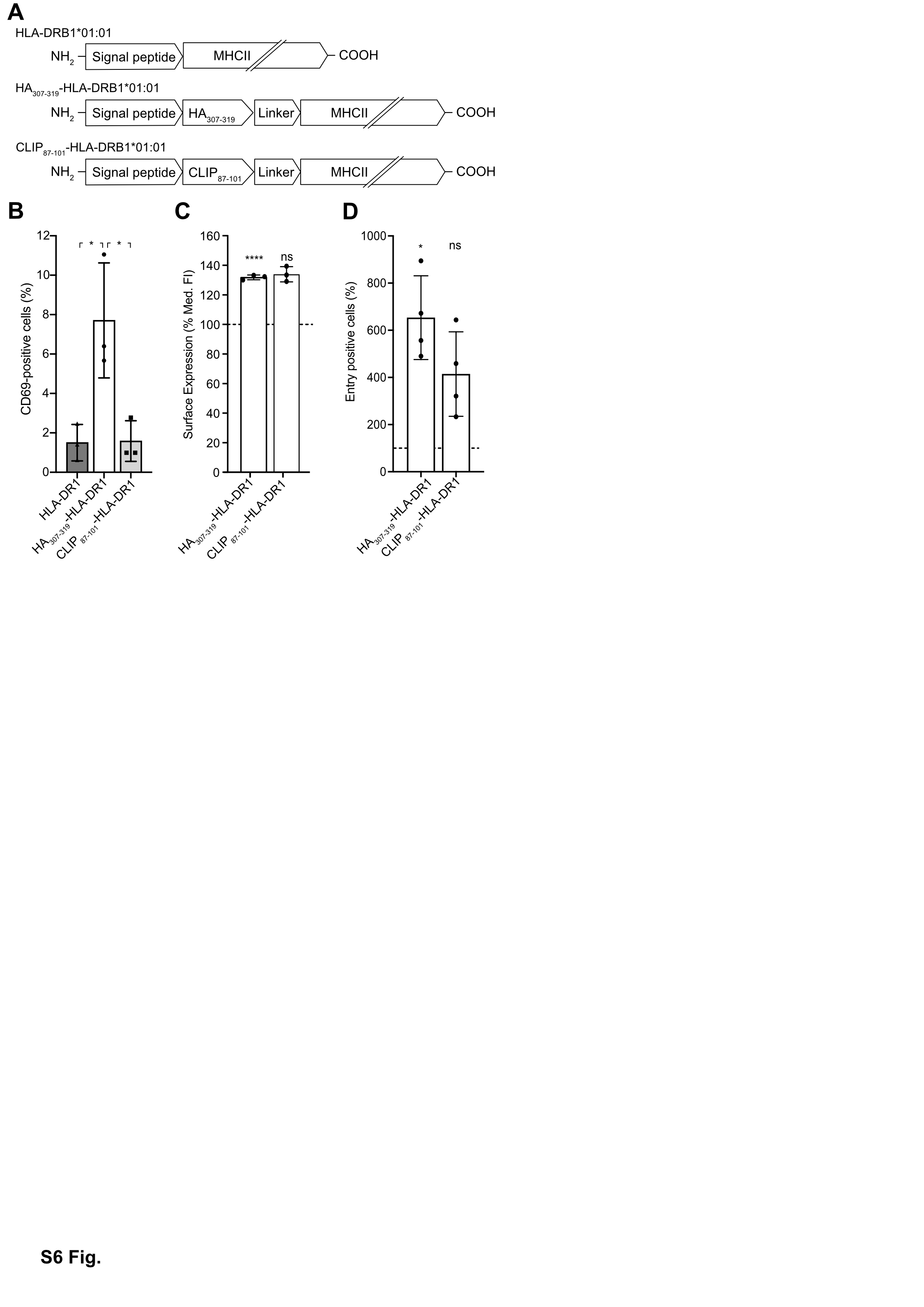

Supplement: S6 Fig — (A) Schematic representation of the DNA construct design used for the expression of MHC-II β-chains (HLA-DRB1*01:01) having the HA307-319 peptide or the CLIP87-101 peptide covalently fused to the N-terminus. (B) T cell activation by BHK21 cells transiently expressing the indicated HLA-DR1 complexes. Underlying data: S6 Data. (C) Flow cytometric analysis of surface expression of the indicated HLA-DR1 complexes. Data were normalized to the surface levels of HLA-DR1 without a covalently fused peptide (dashed line). Underlying data: S6 Data. (D) Susceptibility of cells expressing the indicated HLA-DR1 complexes to VSV-H18 infection. Data were normalized to the susceptibility of cells expressing HLA-DR1 without a covalently fused peptide (dashed line). Underlying data: S6 Data. For statistical analysis, unpaired Student t test was performed for panel B, and one-way ANOVA followed by Dunnett test was used for panels C and D. * P < 0.05, ** P < 0.01, *** P < 0.001, ns, not significant. MHC-II, major histocompatibility complex class II. (TIFF) [file pbio.3002182.s007.tiff]

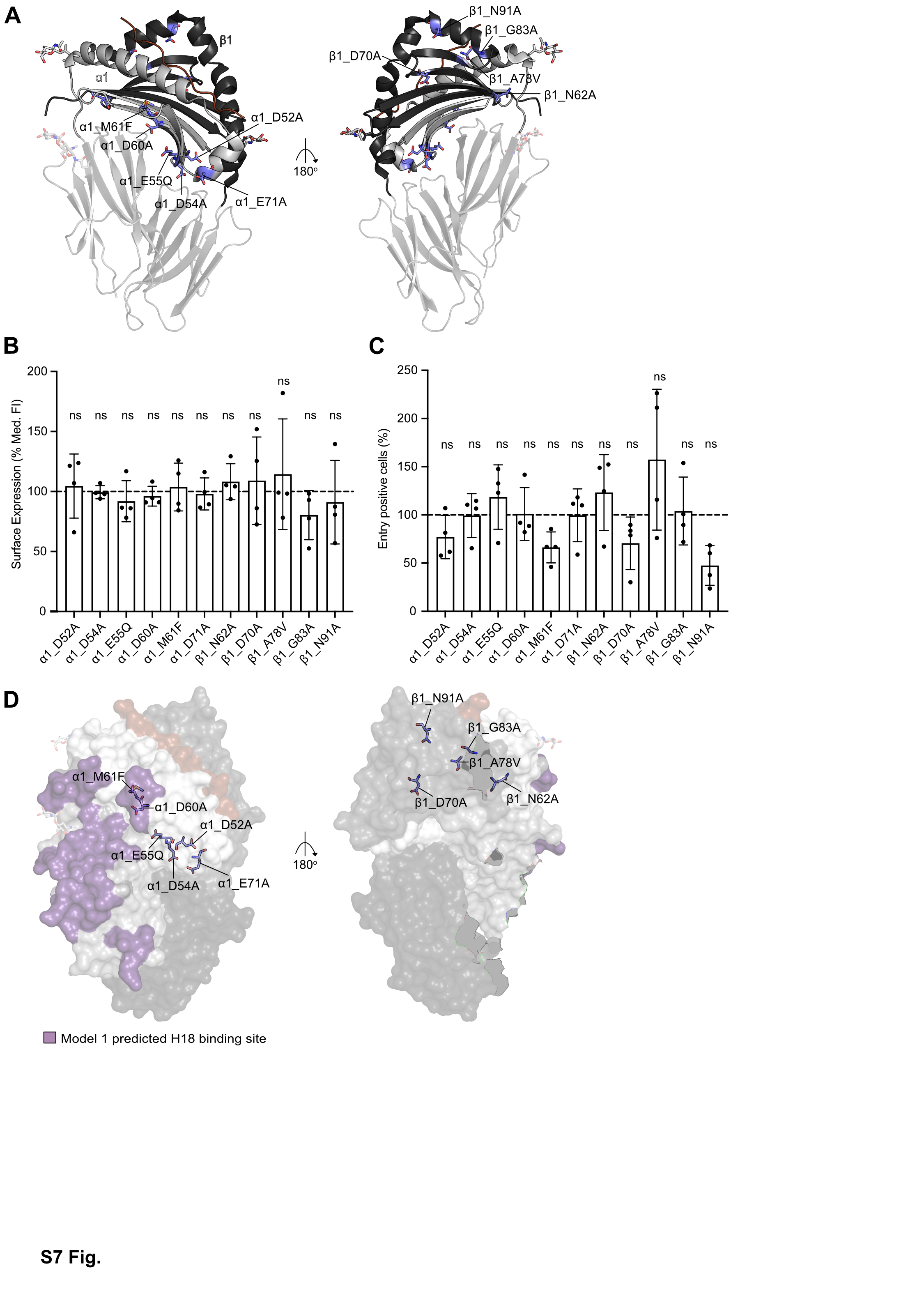

Supplement: S7 Fig — (A) Localization of surface-exposed amino acids in the α1 (gray) and β1 (black) domains of HLA-DR that are present in 75% of all MHC-II homologs and have a conservation score >8. These amino acids were substituted with their HLA-DM counterparts or alanine when conserved. (B) Surface expression of the indicated HLA-DR variants on transfected HEK293T cells. The bar graph depicts the median fluorescent intensity of the β-chain determined from cells that fall into the HLA-DR α-chain and HLA-DR β-chain double positive gate. Values were normalized to wild-type HLA-DR (dashed line). Underlying data: S7 Data. (C) VSV-H18 infection rate of cells expressing the indicated HLA-DR variants depicted in (B). Underlying data: S7 Data. (D) Localization of the highly conserved amino acids substituted with their HLA-DM counterparts or alanine shown in (A) in relation to the predicted H18 binding of model 1 (see also Fig 2H). M61 and D60 are part of the modeled H18 binding surface. For statistical analysis, one-way ANOVA followed by Dunnett test was performed for panels B and C. ns, not significant. HEK293T, human embryonic kidney 293T; HLA-DR, human leukocyte antigen DR; MHC-II, major histocompatibility complex class II. (TIFF) [file pbio.3002182.s008.tiff]
